# TN-GAN-Based Pet Behavior Prediction through Multiple-Dimension Time-Series Augmentation

**DOI:** 10.3390/s23084157

**Published:** 2023-04-21

**Authors:** Hyungju Kim, Nammee Moon

**Affiliations:** Department of Computer Science and Engineering, Hoseo University, Asan-si 31499, Republic of Korea; kimhyungju01@gmail.com

**Keywords:** text-to-numeric generative adversarial network (TN-GAN), behavioral prediction, data augmentation, deep learning

## Abstract

Behavioral prediction modeling applies statistical techniques for classifying, recognizing, and predicting behavior using various data. However, performance deterioration and data bias problems occur in behavioral prediction. This study proposed that researchers conduct behavioral prediction using text-to-numeric generative adversarial network (TN-GAN)-based multidimensional time-series augmentation to minimize the data bias problem. The prediction model dataset in this study used nine-axis sensor data (accelerometer, gyroscope, and geomagnetic sensors). The ODROID N2+, a wearable pet device, collected and stored data on a web server. The interquartile range removed outliers, and data processing constructed a sequence as an input value for the predictive model. After using the z-score as a normalization method for sensor values, cubic spline interpolation was performed to identify the missing values. The experimental group assessed 10 dogs to identify nine behaviors. The behavioral prediction model used a hybrid convolutional neural network model to extract features and applied long short-term memory techniques to reflect time-series features. The actual and predicted values were evaluated using the performance evaluation index. The results of this study can assist in recognizing and predicting behavior and detecting abnormal behavior, capacities which can be applied to various pet monitoring systems.

## 1. Introduction

Behavioral prediction models use statistical techniques, such as algorithm clustering, data mining, or data visualization, to identify and predict object behavior using a machine model or system based on various data collected from video, voice, or sensor movement recordings. Studies of behavioral prediction using image data have limitations because they are sensitive to shooting angle or image quality [1,2]. However, sensor data-based behavioral prediction has been studied more actively because it has relatively fewer limitations and is more cost-effective than image-based research [3]. Behavioral prediction models based on sensor data also use accessible everyday data from automobiles or smart devices (cell phones, tablets, and watches) to make predictions [3,4]. The rapid development of wireless sensor networks has facilitated the collection of numerous data from various sensors [5], and sensors for behavioral prediction include object, environmental, and wearable sensors [6].

An object sensor plays a crucial role in object detection and can infer related behavior after detecting movement. A user attaches a sensor to an object to analyze the object pattern. For example, radio frequency identification automatically identifies and tracks tags attached to objects or furniture and can be used to monitor human or animal behavior.

An environmental sensor is a monitoring system or an internet of things (IoT)-based smart environment application that detects environmental parameters, such as temperature, humidity, and illumination. Environmental sensors play a secondary role in identifying behavior.

Accelerometer and gyroscope sensors are integrated into a wearable built-in devices in smartphones and smartwatch. These sensors are low in cost compared with other sensors. Moreover, the data collected from these sensors are time-series data and can be used for object detection. Additionally, they respond to inputs in the physical environment. Additionally, sensors have applications in various fields. Accordingly, some studies have proposed the development of algorithms or learning models for time-series data analysis [7,8].

The number of pets has recently increased due to the coronavirus disease 2019 (COVID-19) [9], and various pet healthcare products have been released as the pet care market has expanded. Particularly, pet care services using wearable devices have been introduced, and studies on the behavioral recognition of pets have been conducted [10,11,12]. However, such devices have lower accuracy than those used in previous studies on humans. In addition, pets are relatively unable to communicate, making it difficult to obtain data on desired behavior.

Behavioral prediction is based on nine-axis sensors, not the three- or six-axis sensors primarily applied in previous studies. This paper proposes that researchers predict pet behavior using nine-axis sensor data. A device is created for sensor data collection, and the bias of behavioral data is mitigated using the augmented model proposed in this paper. We predict nine behaviors based on this.

## 2. Related Work

### 2.1. Time-Series Data Augmentation

Data augmentation generally improves model performance by increasing the learning data to ensure good results with insufficient or noisy data. Image data are most commonly applied, but time-series data can also be used and are largely divided into data augmentation based on statistical or deep learning methods.

Statistical-based methods include jittering, rotation, permutation, scaling, and other techniques. Random transformation techniques assume that the results do not apply to all datasets. However, pattern mixing, creating a new pattern by combining one or more patterns with existing training data, can combine similar patterns and obtain reasonable results [13].

Deep learning-based data augmentation methods primarily use GAN-based generation models [14]. The GAN was developed in various forms, such as the deep CGAN, CGAN, and super-resolution GAN, to augment data efficiently. Among them, CGAN can augment data for specific classes, and conditional data corresponding to the class are input together. Smith et al. proposed the time-series GAN consisting of two GANs, one regular and one conditional, for 1D time series data [15]. The time-series GAN was applied to 70 time-series datasets, confirming that performance improved for all datasets. Ehrhart et al. proposed a data augmentation technique based on the long short-term memory (LSTM) fully convolutional network CGAN architecture to detect moments of stress [16]. The data were augmented by changing the architecture of the GAN to make it appropriate for research, which performs well.

Therefore, we propose a text-to-numeric GAN (TN-GAN) through multidimensional time-series augmentation, which generates numerical data when text is input based on the nine-axis time-series sensor data used as input for behavioral prediction models. We propose a generative model that generates data based on the experimental group most similar to the input text and that augments the data by specifying specific behaviors. Using this approach, we intend to alleviate the data imbalance problem and improve the learning model performance.

### 2.2. Behavior Recognition

#### 2.2.1. Sensor Data-Based Behavior Recognition

Behavior recognition based on sensor data is collected as time-series data [17]. Many previous studies have used the UCI (University of California, Irvine) dataset or WISDM (Wireless Sensor Data Mining) open dataset to perform behavior recognition [18]. At this time, a fixed time for one behavior is calculated, and a sliding window is applied to the data. Most datasets used in previous studies for behavior recognition mainly used accelerometer and gyroscope sensors. Additionally, depending on the purpose, more biosensors for monitoring systems are being added. For example, environmental sensors, such as temperature, humidity, and illumination sensors, are added to collect data.

Even if behavior recognition is performed by applying the same algorithm to the same data set, the result may differ depending on the data preprocessing method or results. Zheng et al. compared the segment length and data conversion methods of open datasets, such as WISDM and Skoda open datasets. The multi-channel method (MCT, Multi-Channel Transformation) used by these authors showed better performance [19].

Additionally, the sensors or modules of the collected data offer higher accuracy in behavior recognition when using multiple sensors than when using single sensors. Generally, only an accelerometer sensor or gyroscope sensor is used with a single sensor. However, a wearable device is configured with multiple sensors. Moreover, multiple sensors are characterized by a module, such as accelerometer, gyroscope, and geomagnetic. Mekruksavanich et al. confirmed that when single-sensor and multi-sensor values were divided, using a smartwatch as input values, the learning results of the same model were better [20].

Thus, we aim to predict the composition of the sensor based on behavior recognition after classifying the behavior, using an accelerometer, gyroscope, and geomagnetic sensor module rather than a single sensor.

#### 2.2.2. Deep Learning-Based Behavior Recognition

Monitoring systems are studied for behavioral recognition and pattern analysis applied to humans in real life, and behavioral recognition performs well due to the development of artificial intelligence [21]. Existing recognition approaches include machine learning (e.g., the decision tree, random forest, support vector machine, naive Bayes, and Markov models). A machine learning algorithm has been used to achieve human activity recognition [22,23]. However, this method is inefficient and time-consuming because it involves many preprocessing steps and inefficient operations.

Deep learning is a structure created by imitating human neural networks. Deep learning technology in artificial neural networks has been developed to solve machine learning problems, and learning methods that can be applied in various ways by learning features have been studied. Convolutional neural networks (CNNs) and recurrent neural networks (RNNs) have been devised as representative learning methods. CNNs perform excellently in image processing and preserve spatial structural information, with 1D vector values being used as learning input. Although learning takes a long time due to the numerous nodes within the hidden layer, the shortcomings have been supplemented with parallel computing operations using graphics processing units (GPUs) [24].

RNNs are artificial neural network models in which the previous calculation results influence the current output of cells. In a general deep neural network structure, values with an activation function proceed toward the output layer. However, an RNN sends data from the nodes of the hidden layer to the output and hidden layers. Thus, continuous information can be reflected; however, a dependency problem occurs when the dependence period becomes long. The LSTM method was developed to solve this problem [25,26] and was designed for long-term dependency on related information by adding cell statesf to the existing RNN model. Therefore, LSTM methods are used in various fields in time-series data-based research [27]. CNN and LSTM method are predominantly employed because each CNN extracts features from deep learning-based behavioral recognition, and because the LSTM reflects time-series characteristics.

### 2.3. Comparison with Previous Studies

Most studies on human behavioral recognition have used open datasets, and various studies have been conducted on preprocessing and model construction. Therefore, research on behavioral recognition based on sensor data is not limited to humans but has also been conducted on pets and livestock. Table 1 compares previous studies on sensor data-based behavioral recognition. This study contributes to the existing research on behavioral prediction based on nine- and three-axis sensor data that previously recognized and predicted behavior. We predict pet behavior using a hybrid deep-learning CNN and LSTM model that outperformed machine learning. Additionally, data augmentation removed bias to improve performance, and the prediction was made after a nine-behavior classification.

## 3. CNN-LSTM-Based Behavior Prediction with Data Augmentation

**Figure 1:**
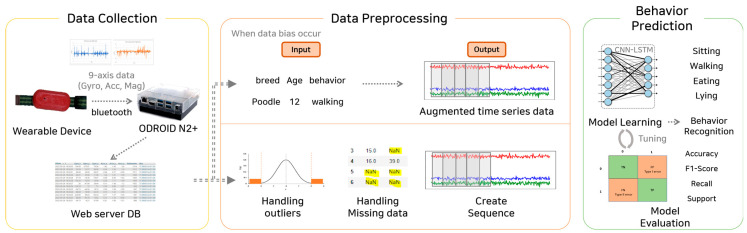
Behavioral prediction processes through multidimensional time−series augmentation.

This study proposes deep learning-based behavioral prediction through multidimensional time-series augmentation, grouped into data collection, preprocessing, and behavioral prediction. Figure 1 illustrates the entire process.

After collection, the nine-axis sensor data (accelerometer, gyroscope, and geomagnetic sensors) and image data were stored in the data collection device using Bluetooth communication. Afterward, the saved data were transmitted to the web server database, in which sequences were used as input values for the learning models, and processed to detect outliers and missing values. When data biases occurred, time-series sensor data were augmented using the data augmentation model for the text data, such as the breed, size, and behavior. Then, performance was verified using the trained deep learning CNN-LSTM model. Moreover, the actual labeled behavioral data and predicted values were compared using the performance verification index.

### 3.1. Data Preprocessing

#### 3.1.1. Remove Outliers

Outliers were removed using the interquartile range (IQR), the difference between the values at 25% and 75%. The IQR is commonly used to remove outliers because it sorts data in ascending order and divides datasets into four equal parts. The data is divided into the quartiles of 25%, 50%, 75%, and 100% of the data. After determining the IQR, we multiplied it by 1.5 and added the result to the 25% value to determine the minimum value and to the 75% value to determine the maximum value. Values which were smaller or larger than the determined minimum or maximum values, respectively, were outliers.

#### 3.1.2. Data Normalization

Normalization refers to a change in the range of values on a common scale without distorting differences in the range by adjusting them. The z-score normalization was performed on the dataset, a method in which the IQR removes outliers. The z-score refers to changing the value corresponding to the standard normal distribution, and is calculated using the standard deviation and mean value.

#### 3.1.3. Missing Value Interpolation

A missing value occurs when no value exists in the data after data collection. Substantial existing data are lost if missing values are not processed or if interpolation is not performed. This study employed the cubic spline interpolation to address missing value problems. Cubic spline interpolation interpolated a curve, connecting two points using a cubic polynomial, and was performed in sequences where the rate of missing sequences that constituted a behavior was 10% or less.

#### 3.1.4. Creating Sequences

It is vital to create data sequences as the input to the learning model. A sequence is created by estimating 3 s behavior based on the previously processed outliers, normalization, and missing value-interpolated data. The sensor data frequency was 50 Hz; thus, the length of one data sequence corresponding to 3 s comprised a sequence of 150 s. The constructed dataset comprised a three-axis accelerometer, three-axis gyroscope, and three-axis geomagnetic sensor. Thus, nine data sequences were created, consisting of 150 s. A sliding window was applied to the generated sequences, with a ratio of 50%.

### 3.2. TN-GAN-Based Multidimensional Time-Series Augmentation

We propose a multidimensional time-series data augmentation method based on the TN-GAN. The establishment of the model structure began with the word embedding part of stackGAN, which has a structure that creates an image when text is entered. This paper presents a model structure that fuses the upsampling of stackGAN and the downsampling process of a general GAN.

Data were generated using a generative model to augment multidimensional time-series data. The text data were received as input values, and the input behavioral data were augmented using word embedding based on the experimental group which was most similar to the input value. The input values included gender, age (in months), breed, weight, and behavior. Then, these variables were converted into dense vectors using word2vec. Weight values were adjusted on variables that affected pet activity after establishing internal criteria based on veterinary papers [34,35]. A vector value was calculated based on weights of 0.5 for the breed, 0.4 for age, and 0.1 for gender. The behavioral input was augmented based on the sum of the embedding vectors calculated through word2vec based on the most similar experimental group. Behaviors were converted into one-hot vectors to classify input behaviors separately. Afterward, data augmentation proceeded by receiving the biased behavior or the behavior requiring augmentation as the last input value.

**Figure 2:**
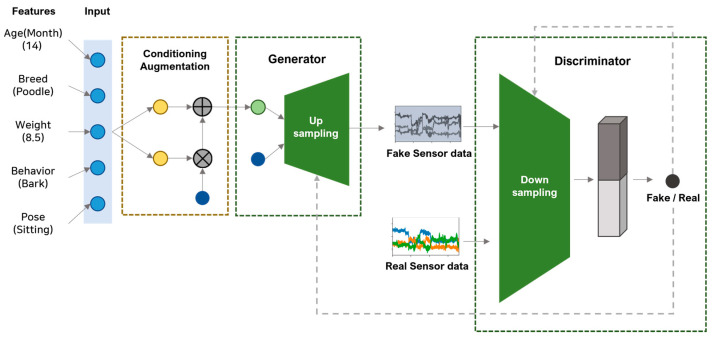
Data generation process of the TN-GAN.

**Figure 3:**
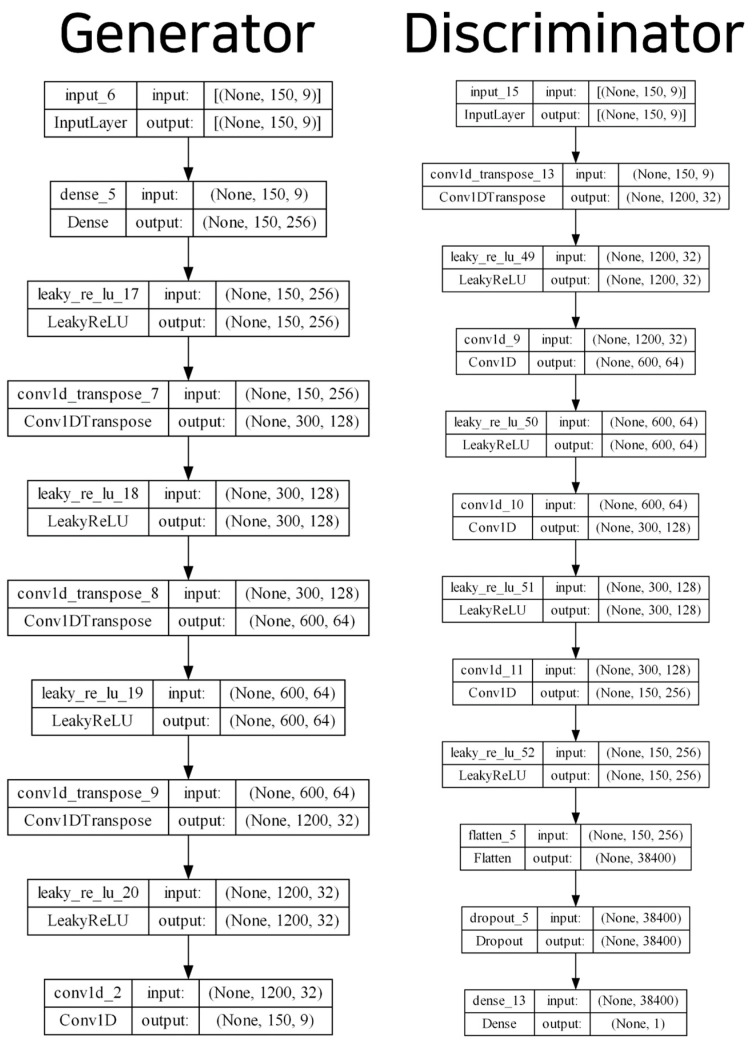
Generator and discriminator hyperparameters and model structure of the TN-GAN model.

Figure 2 presents the overall process of the generative model, and Figure 3 displays the model structure of the generator and discriminator of the TN-GAN. The input layer of the generator model comprised (150, 9); we derived these values based on sensor data lengths of one sequence of 150 s and the gyroscope, accelerometer, and geomagnetic field values, consisting of x, y, and z, respectively. Then, the upsampling structure of the stackGAN was implemented using the stacking 1D CNN layers. The leaky rectified linear units (ReLU) activation function was used, and fake sensor data were created in the form of (150, 9). The discriminator model received data in the form of (150, 9) and was created using a generator that passed through a 1D CNN layer and leaky ReLU with a downsampling structure. Finally, the neurons were spread through the flattened layer and, using the dense layer and softmax function, the discriminator model determined whether the generated data were fake or real with a value from 0 to 1.

### 3.3. Pet Behavior Prediction Model

**Figure 4:**
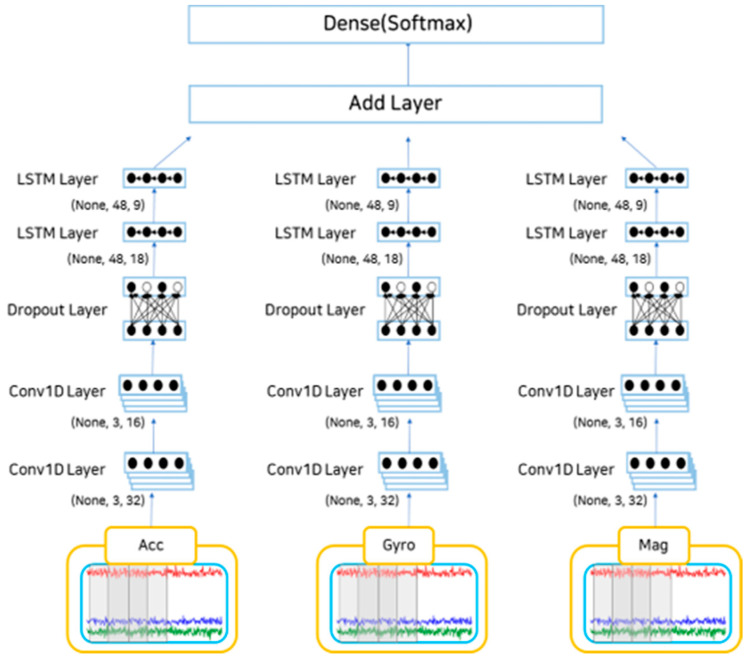
CNN-LSTM hybrid model of the behavioral prediction model structure.

Behavioral recognition was performed on a dataset that had undergone preprocessing and data augmentation. In this study, the behavioral recognition model comprised a 1D form of the CNN-LSTM hybrid model designed to reflect the characteristics of behavioral recognition patterns, and LSTM reflected time-series features. Figure 4 illustrates the structure of the behavioral recognition model.

The sensor data employed as input values were not calculated immediately but were divided into values from a three-axis accelerometer, three-axis gyroscope, and three-axis geomagnetic sensor. After receiving each set of three-axis data as input values and applying a 1D CNN layer, the size of the existing filter was reduced by half, and the CNN of the downsampling process was performed again. After passing the dropout layer to prevent overfitting, the LSTM layer proceeded in the same way as in the CNN. The layer was added after completing the calculation up to the LSTM layer for each sensor. The dense layer for multiclassification and behavior was classified using the softmax function, and the performance was measured through indicators for the predicted behavior based on the actual behavior.

## 4. Experiments

### 4.1. Experimental Setup

This study was implemented using Keras as the backend and TensorFlow in Python. Table 2 lists the detailed specifications of the experiment.

### 4.2. Data Collection and Dataset

The participants were recruited for data collection, and data were collected on 10 pets. The data collection environment was set indoors, and data were only collected in an environment where a companion was with the pet so that it was not anxious. Basic information on the dogs is presented in Table 3.

**Figure 5:**
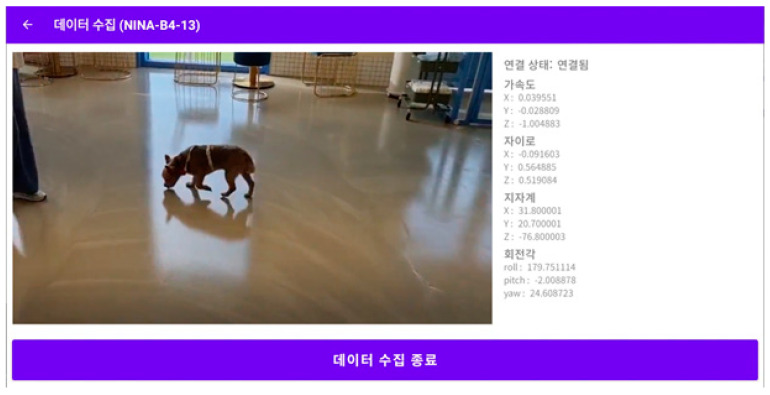
Application data collection screen. 데이터 수집: Data Collection. 연결 상태: 연결됨 Status: connect. 가속도: accelerometer. 자이로: gyroscope. 지자계: geomagnetic. 회전각: angle of rotation. 데이터 수집 종료: End Data Collection.

**Figure 6:**
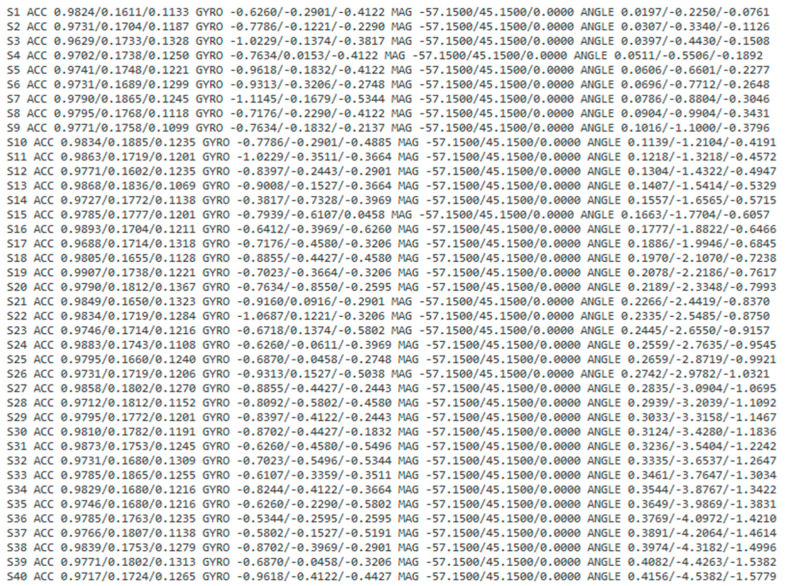
Data collection console screen using Eclipse Maxim SDK.

**Figure 7:**
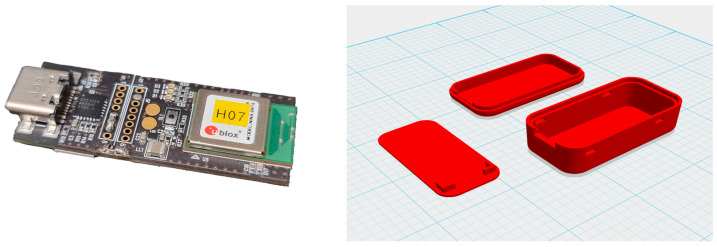
(**Left**) Printed circuit board. (**Right**) Device case.

**Figure 8:**
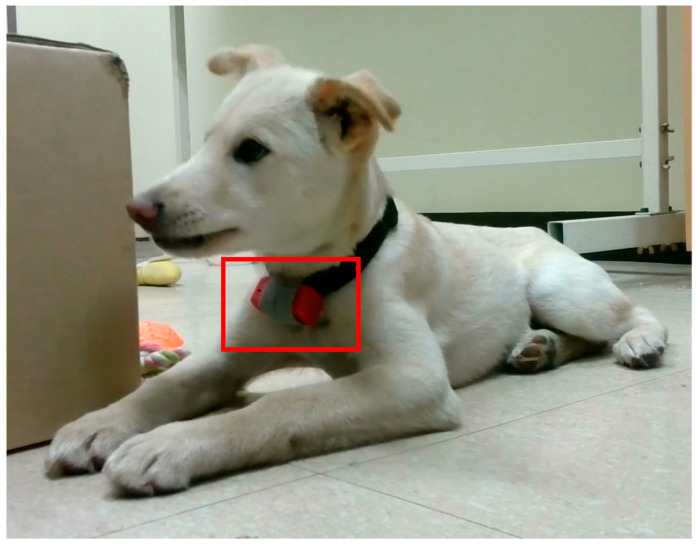
Wearable device example in the red rectangle on a dog.

The data collection screen in the ODROID application is shown in Figure 5, and the console screen of the collected data is shown in Figure 6. The wearable data collection device was fabricated using a nine-axis sensor-based printed circuit board (PCB). Its weight was approximately 28 g, excluding the collar, and the case was created using a 3D printer. Figure 7 provides the images of the board and case, and Figure 8 depicts the worn example. The frequency of the sensor data was 50 Hz, and the device was developed using the Eclipse Maximum SDK.

A total of 26,912 data were collected through the process outlined. The number of recognized behaviors was nine, and Table 4 lists the data distribution for each behavior.

### 4.3. Data Preprocessing

**Figure 9:**
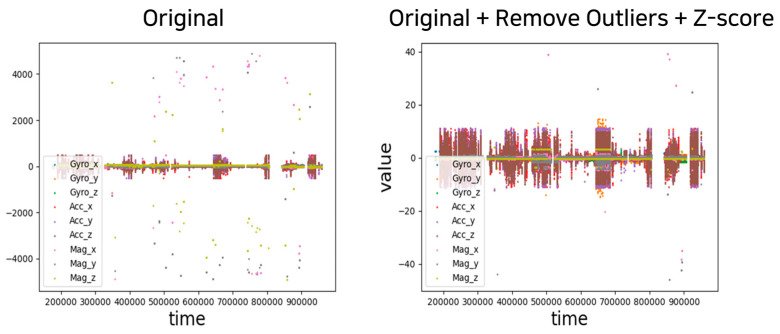
Comparison graph before and after sensor data preprocessing.

Outlier removal and data normalization were performed based on the collected nine-axis sensor dataset. Figure 9 illustrates the original data and results after preprocessing. Then, cubic spline interpolation was applied to the preprocessed training data. The standard for one behavior was calculated as 3 s, and the sensor data were collected at 50 Hz; thus, the length of sensor data constituting one sequence was 150 s. Interpolation was performed for sequences in which 10% of the length had fewer than 15 missing values.

**Figure 10:**
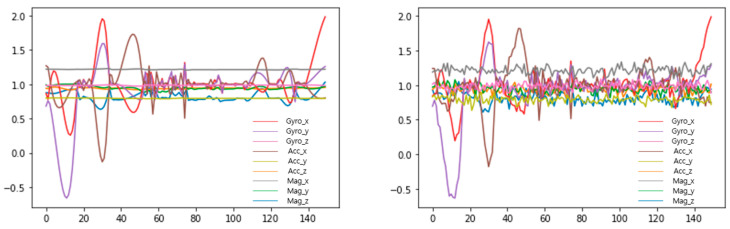
Behavioral data (walking) augmented graph ((**Left**): original, (**Right**): augmented).

Multidimensional sensor data were augmented by using the TN-GAN to mitigate data bias. Instead of removing behaviors with numerous data, reinforcement was performed for behavior with insufficient data. When there were 500 or fewer data, the data were augmented by a factor of 3, and 1000 data or fewer were augmented by a factor of 2. Figure 10 presents the graph before and after augmentation, and Table 5 displays the dataset distribution that merges the original and augmented data through the TN-GAN.

### 4.4. Behavior Prediction Model Learning

The Adam optimizer was applied as an optimization function for behavioral prediction, and the learning rate was set to 0.001. The batch size for learning was set to 4, and overfitting was prevented using early stopping as a callback function. Learning was iterated 200 times. The leaky ReLU was the activation function in the CNN layer, and the LSTM layer applied the hyperbolic tangent activation function. Performance indicators were measured based on precision, F1-score, and recall values. Table 6 presents the experimental results for behavioral prediction.

**Figure 11:**
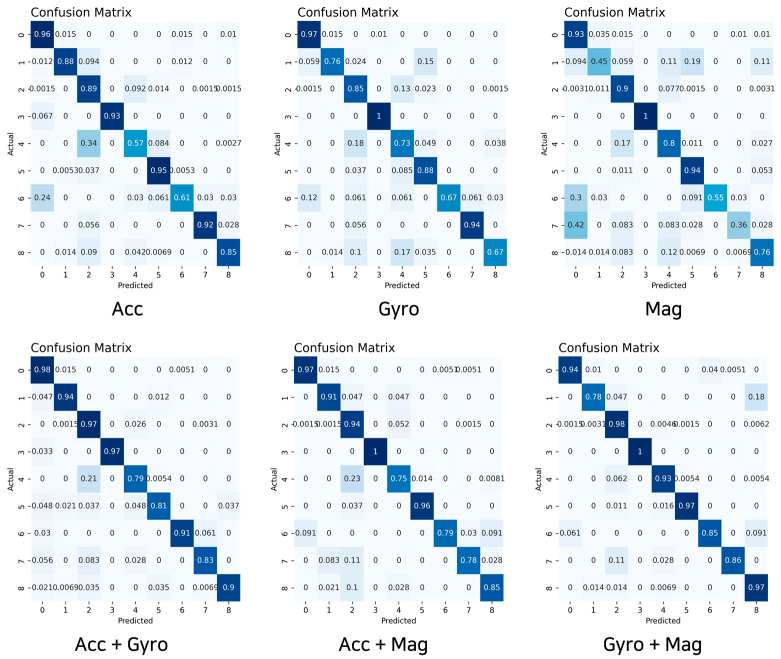
Behavioral prediction confusion matrix using three- and six-axis data.

**Figure 12:**
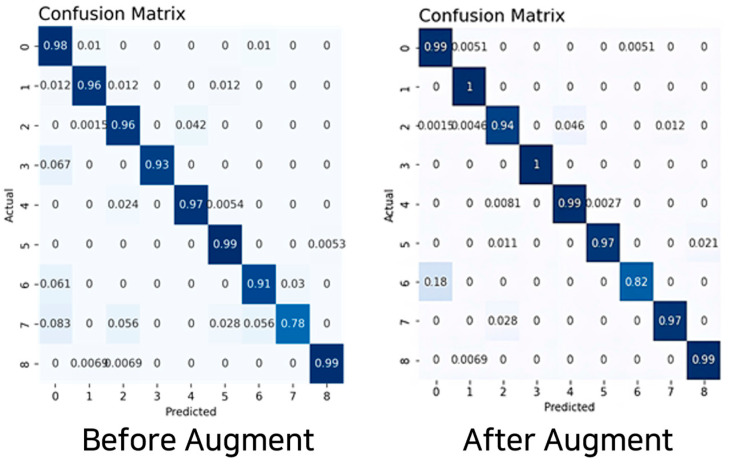
Behavior prediction confusion matrix using the nine-axis sensor data.

The experimental results reveal that nine-axis sensor data using the accelerometer, gyroscope, and geomagnetic sensors performed the best. The augmented dataset using the TN-GAN model displayed the highest accuracy at 97%. All training models used a CNN-LSTM hybrid model, and the results indicated that the best performance was attained on the basis of hyperparameter tuning rather than using the same hyperparameters for each dataset. The result of the behavioral prediction accuracy, attained based on the CNN-LSTM model and derived using the augmented nine-axis sensor data, was 97%; the recall and F1-score values were the same. Table 7, Figure 11 and Figure 12 provide the prediction results for behavioral classification.

High precision, recall, and F1-score values were obtained for all behaviors. The behaviors “sitting on four legs” and “standing on four legs” demonstrated high predictive results. The behavior with the lowest predictive result was “walking.” This can be explained by the fact that a dog, upon stands up onto its feet, jumps either toward its owner or jumps towards a wall or object for support, confusing the behavioral prediction process.

## 5. Conclusions

This paper proposes predicting the behavior of pets using nine-axis sensor data (accelerator, gyroscope, and geomagnetic sensors). Nine behaviors (standing on two legs, standing on four legs, sitting on four legs, sitting on two legs, lying on the stomach, lying on the back, walking, sniffing, and eating) were assessed for prediction, and the standard length of time for each was 3 s.

The experimental group in the dataset consisted of 10 animals. Wearable devices were manufactured using a PCB and 3D printers, and data were stored and transmitted using ODROID N2+. The data collection frequency was 50 Hz. We aimed to demonstrate the high performance of the collected sensor data by using them as input values for the prediction model through preprocessing processes, such as in outlier processing, missing value interpolation, and data normalization processes.

In the event of data bias, we aimed to augment the data through the TN-GAN-based multidimensional time-series data generation model proposed in this paper. The generation model received text data as input values and embedded them to augment the behavioral data through the GAN. This was done on the basis of the experimental group most similar to the data specified in the input values.

Based on the sensor dataset with the bias removed via augmentation, a sequence for the learning model was constructed for use as an input value. The experiment was conducted with three-, six-, and nine-axis sensor data. The behavioral prediction was conducted using the CNN-LSTM hybrid model. The nine-axis sensor data were compared before and after augmentation.

The experimental results revealed that the augmented nine-axis sensor data performed best, with a score of 97%, displaying excellent performance in behaviors other than walking. Moreover, when data bias occurred, numerous learning data could be used because they were augmented without adjusting the class weight or removing high-weight data.

In future research, we intend to recognize and predict more behaviors than in the existing experiments by improving the recognition rate of dynamic behavior. In situations when the existing daily behavioral prediction displays high levels of performance, we aim to detect and predict abnormal behavior. Therefore, we aim to expand on previous studies in order to assess more diverse companion animal monitoring systems.

## Figures and Tables

**Table 1:** Comparison of research on behavioral recognition based on sensor data.

No.	Sensor Data	Device Position	Frequency (Hz)	Number of Behaviors	Suggested Model
[20]	Gyro +Acc	Wrist	20	18	CNN-LSTM
[28]	Acc	Neck + leg	10	4	Random Forest
[29]	Gyro + Acc	Neck + Back	100	7	SVM, Decision Tree
[30]	Gyro + Acc	Neck + Tail	33.33	10	CNN
[31]	Acc	Neck + Back	50	8	LSTM
[32]	Acc	Neck	25~50	9	FilterNet
[33]	Acc + Mag	Neck	12.5	7	KNN, Random Forest
Proposed	Gyro + Acc + Mag	Neck	50	9	CNN-LSTM

Note: Gyro: gyroscope; Acc: accelerometer; Mag: geomagnetic sensor; CNN: convolutional neural network; LSTM: long short-term memory; SVM: support vector machine; KNN: k-nearest neighbors.

**Table 2:** Experimental specifications.

Metric	Description
CPU	AMD Ryzen 5800X
GPU	NVIDIA RTX 3090(2way)
RAM	64 GB
CUDA	11.1
cuDNN	8.1
Python	3.9.6
TensorFlow	2.10.0

**Table 3:** Basic experimental subject information.

No	Breed	Age (Month)	Weight (Kg)
1	Yorkshire terrier	60	8
2	Toy poodle	76	4.7
3	Toy poodle	150	5
4	Toy poodle	130	1.5
5	Mini poodle	68	2.2
6	Mini bichon	94	3.1
7	Mix dog	36	4.6
8	Mix dog	42	4.2
9	Border collie	24	14.5
10	Mix dog	12	6.3

**Table 4:** Experimental dataset configuration.

No. (Label)	Behavior	Number of Data
0	Standing on two legs	434
1	Standing on four legs	4769
2	Sitting on two legs	7179
3	Sitting on four legs	6492
4	Lying on the stomach	2524
5	Lying on the back	1277
6	Walking	1529
7	Sniffing	774
8	Eating	1934
Total		26,912

**Table 5:** Configuration before and after experimental dataset augmentation.

No. (Label)	Behavior	Number of Data (before Augment)	Number of Data (after Augment)
0	Standing on two legs	434	1302
1	Standing on four legs	4769	4769
2	Sitting on two legs	7179	7179
3	Sitting on four legs	6492	6492
4	Lying on the stomach	2524	2524
5	Lying on the back	1277	1277
6	Walking	1529	1529
7	Sniffing	774	1548
8	Eating	1934	1934
Total		26,912	28,554

**Table 6:** Performance results for each dataset.

Dataset	Precision	Recall	F1-Score
Acc	0.78	0.80	0.79
Gyro	0.77	0.83	0.80
Mag	0.64	0.68	0.66
Acc + Gyro	0.91	0.93	0.92
Acc + Mag	0.91	0.91	0.91
Gyro + Mag	0.91	0.93	0.92
Acc + Gyro + Mag (Before Augment)	0.94	0.95	0.95
Acc + Gyro + Mag (After Augment)	0.95	0.97	0.96

**Table 7:** Behavioral prediction model of the training results.

No. (Label)	Behavior	Precision	Recall	F1-Score
0	Standing on two legs	0.98	0.99	0.98
1	Standing on four legs	0.94	1.00	0.97
2	Sitting on two legs	0.99	0.94	0.96
3	Sitting on four legs	1.00	1.00	1.00
4	Lying on the stomach	0.92	0.99	0.96
5	Lying on the back	0.99	0.97	0.98
6	Walking	0.93	0.82	0.87
7	Sniffing	0.81	0.97	0.89
8	Eating	0.97	0.99	0.98

## Data Availability

The data presented in this study are available from the corresponding author upon request. The data are not publicly available due to privacy and ethical concerns.
